# Cross‐Ancestry Polygenic Prediction: Comparing Methods and Assessing Transferability Across Traits

**DOI:** 10.1002/gepi.70029

**Published:** 2026-01-21

**Authors:** Md. Moksedul Momin, Xuan Zhou, Muktar Ahmed, Elina Hyppönen, Beben Benyamin, S. Hong Lee

**Affiliations:** ^1^ Australian Centre for Precision Health University of South Australia Adelaide Australia; ^2^ UniSA Allied Health and Human Performance University of South Australia Adelaide Australia; ^3^ Department of Genetics and Animal Breeding, Faculty of Veterinary Medicine Chattogram Veterinary and Animal Sciences University (CVASU), Khulshi Chattogram Bangladesh; ^4^ South Australian Health and Medical Research Institute (SAHMRI) Adelaide Australia; ^5^ UniSA Clinical and Health Sciences University of South Australia Adelaide Australia

## Abstract

Accurate prediction of disease risk and other complex traits across different populations is essential for clinical and research purposes. However, genetic differences among ancestries, such as allelic frequencies and genetic architecture, can affect the performance of polygenic risk score (PGS) methods in cross‐ancestry prediction. To address this issue, we conducted a formal test of seven polygenic prediction methods applicable across ancestries for five traits (BMI, standing height, LDL‐, HDL‐ and total‐cholesterol) from the UK Biobank dataset. We demonstrate that, GBLUP and PRS‐CSx outperformed other methods for highly polygenic traits like height and BMI. In contrast, PRSice and PolyPred performed best for less polygenic traits like cholesterol, with PRS‐CSx being comparable with larger sample sizes. We also observed that utilizing concordant SNPs, which have the same effect direction across diverse ancestries, can improve the accuracy of cross‐ancestry PGS models. Furthermore, we found that the transferability of PGS across ancestries varied depending on the trait. Understanding the strengths and limitations of different methods and approaches is important for future methodological development and improvement, enabling better interpretation and application of PGS results in clinical and research settings.

## 1 Introduction

Human traits are often influenced by multiple genes, resulting in a complex inheritance pattern that is not easily predictable. This phenomenon, known as polygenic inheritance, and is believed to be a combination of genetic variation and environmental factors (Favé et al. 2018; Momin et al. 2023a). For many complex traits and diseases with significant public health implications, hundreds or even thousands of genetic variants or polymorphisms are involved, each having a small effect on disease risk (Lewis and Vassos 2020). Some traits, such as standing height and BMI, are highly polygenic, while others, like HDL and LDL cholesterol levels, are less polygenic (Snieder et al. 1999) and primarily controlled by a smaller number of genes which each have a relatively large effect (major genes).

Genome‐wide association studies (GWAS) have made significant progress in identifying tens of thousands of genetic variants associated with complex traits and diseases, primarily in European ancestries (Bustamante et al. 2011; Popejoy and Fullerton 2016). While many of these associations have been replicated in other ancestry groups (Waters et al. 2010; Carlson et al. 2013; Hindorff et al. 2009), that is, are believed to be concordant, the extent to which findings from White European populations can be extrapolated to other populations remains uncertain in human genomics. Moreover, the overrepresentation of European populations in GWAS can lead to a bias towards identifying genetic variants that are more common in European populations and may miss genetic variants that are specific to or more prevalent in other populations. This can limit the generalizability and transferability of GWAS results to non‐European populations, as well as potentially reinforce health disparities between different ethnic groups (Martin et al. 2017).

Polygenic risk scores (PGS) are estimates of an individual's likelihood or risk for complex traits or diseases based on their genetic profile, and their accuracy increases when predictions are based on sufficiently large GWAS discovery samples (Ni et al. 2021). PGSs can potentially provide insights into future health outcomes and inform diagnostic and preventive strategies (Ruan et al. 2022). However, recent studies have shown that PGSs have significantly lower prediction accuracy when the training and target samples are genetically diverse, that is, cross‐ancestry prediction (Martin et al. 2017; Ruan et al. 2022; Martin et al. 2019). The majority of GWASs to date have been conducted in individuals of European descent (Hindorff et al. 2018; Peterson et al. 2019), and there are many genetic studies to show that the accuracy of PGSs based on European training data is lower when applied to non‐European populations (Martin et al. 2019; Peterson et al. 2019; Márquez‐Luna et al. 2017; Duncan et al. 2019; Sirugo et al. 2019). The decreased accuracy can be attributed to differences in linkage disequilibrium (LD) (Ruan et al. 2022; Sirugo et al. 2019), changes in allele frequency (some SNPs are population‐specific) (Martin et al. 2019; Wang et al. 2020a; Durvasula and Lohmueller 2021), and changes in causal effect sizes (Martin et al. 2019; Wang et al. 2020a; Kuchenbaecker et al. 2019), and differences in heritability (Martin et al. 2019; Wang et al. 2020a).

The transferability of PGS from European populations to other ancestries is a growing concern in genomic research, given the lack of sufficient reference samples for non‐European populations. Previous studies have indicated that certain SNPs have stronger effects in specific ancestries, leading to limited effectiveness of cross‐ancestry PGS for those ancestries (Graham et al. 2021). Despite efforts to include non‐European populations in PGS construction, PGS methods still heavily rely on European discovery samples to predict PGS for non‐European target samples, where SNP effects are trained in European discovery samples. Several methods are available (e.g., XPASS [Cai et al. 2021], PolyPred [Weissbrod et al. 2022], PRSice [Euesden et al. 2015], and PRS‐CSx [Ruan et al. 2022]) for constructing cross‐ancestry PGS. However, evaluating the performance of these methods in cross ancestry context can be challenging due to the sampling variance of PGS estimates in target ancestries when using real data. This issue is particularly problematic for non‐European populations because the lack of sufficient sample sizes can make it difficult to use cross validation techniques that can assess the variability of predictors in the target population.

The objective of our study is to determine the most effective cross‐ancestry polygenic prediction method, accounting for varying discovery sample sizes. To achieve this, we conducted a comparison of seven cross‐ancestry genomic prediction methods using real data from the UK Biobank, with a focus on highly polygenic traits such as height and BMI, as well as less polygenic traits like cholesterol. Our evaluation was based on a discovery population of white British, with Other Europeans, South Asians, and Africans as the target populations. To assess the performance of the PGS methods, we utilized the recently developed R‐package r2redux (Momin et al. 2023b). Additionally, we explored whether the transferability of PGS across ancestries varied depending on the trait. Finally, we examined the performance of concordant SNPs versus total SNPs across various traits and methods in cross‐ancestry genetic prediction settings.

## 2 Methods and Materials

### 2.1 Genotype Data and Quality Control

There are 501,748 participants in the UK Biobank (https://www.ukbiobank.ac.uk/) whose data were collected between 2006 and 2010 (Fry et al. 2017). Data collection was conducted in 22 assessment centers in England, Wales, and Scotland, and participants ranged in age from 37 to 73 years (Ollier et al. 2005). In this study, we used the second release of the UK Biobank genotype data, which consists of 92,693,895 imputed autosomal SNPs for 488,377 individuals. The UK Biobank individuals were stratified according to their ancestry using principal component (PC) analysis (Novembre and Stephens 2008) into white British, Other European, South Asian and African following previous approach factors (Momin et al. 2023a, 2024). We retained only HapMap3 SNPs for cross‐ancestry genomic prediction, which are also considered robust and reliable (Tropf et al. 2017; Bulik‐Sullivan et al. 2015). Each ancestry was subjected to stringent quality control (QC) procedures. As part of the SNPs quality control criteria, SNPs excluded with an INFO of < 0.6 (Border et al. 2019; Lee et al. 2017; Peyrot et al. 2018), a call rate of < 0.95, a MAF of < 0.01 and a Hardy‐Weinberg equilibrium *p*‐value of 10^‐4^ are considered. Further, we exclude outliers of the population (individuals outside six standard deviations) and closely related individuals (‐rel‐cutoff 0.05). An individual level QC criterion includes genotype missing rates > 0.05, gender mismatches (reported gender differs from genetically assigned sex), or sex chromosome aneuploidies. Conversely, for BioBank Japan (BBJ) dataset, our access was limited to summary‐level data, which constrained the depth of methodological description we could provide. We chose BBJ specifically because it offers a large, well‐characterized sample with extensive SNP coverage for the traits of interest. This ensures sufficient statistical power for our genetic analyzes and facilitates cross‐population comparisons with the UKB cohort.

### 2.2 Discovery and Target Data

In this study, we used two white British subsets from the UK Biobank, consisting of 50,000 and 258,792 individuals, respectively, as discovery samples for cross‐ancestry genomic prediction in the target datasets. It is important to note that the 50,000 individuals represent a subset of the 258,792 individuals. For all method comparisons, the target datasets consisted of Other European (*n* = 26,457), South Asian (*n* = 6199), and African (*n* = 6179) ancestry groups across methods. Additionally, we had access to GWAS summary statistics for BMI (Akiyama et al. 2017) (sample size = 158,284), standing height (*n* = 159,095) (Akiyama et al. 2019), total cholesterol (Kanai et al. 2018) (*n* = 128,305), HDL‐cholesterol (Kanai et al. 2018) (*n* = 70,657), and LDL‐cholesterol (Kanai et al. 2018) (*n* = 72,866) from the Japanese Biobank (BBJ) (http://jenger.riken.jp/en/result).

### 2.3 Annotation of Concordant SNPs

To identify concordant and discordant SNPs, we compared the direction of SNP effects between two independent GWAS datasets: the white British cohort from the and individuals from Biobank Japan (BBJ). SNPs with strand ambiguity or alignment issues were excluded from the analysis. To classify SNPs as concordant or discordant, we compared the direction of effect alleles across the two populations following Momin et al (Momin et al. 2024). After QC, there were 4,113,630 SNPs common to UKBB and BBJ for BMI, total‐, LDL‐, and HDL‐cholesterol while 3,442,047 SNPs were common for standing height. Of these SNPs, 55% had concordant association on BMI and 52% had concordant effects on cholesterol (Supporting Information Table S1), while the rest had discordant effects. We used white British as the discovery population to predict phenotypes in the target ancestries and performed cross‐ancestry genomic prediction using HapMap3 SNPs from the 4,113,630 SNPs, as they are considered reliable for genetic testing. The total number of common concordant and discordant SNPs between the discovery and target datasets are shown in Supporting Information Table S2.

### 2.4 Phenotypic Data

To compare cross‐ancestry genomic prediction methods, we analyzed five traits from the UK Biobank: two highly polygenic anthropometric traits (BMI and standing height) and three less polygenic cholesterol traits (total‐, HDL‐, and LDL‐cholesterol). To control for potential confounding factors, we adjusted these phenotypes using a linear model function in R‐software (version 4.0.3), which included covariates sex, birth year, education, Townsend deprivation index, assessment center, genotype measurement batch, and population structure (measured by the first 10 principal components). For the information of educational qualifications, we converted the variable into education levels (years), following a previously established approach (Okbay et al. 2016).

### 2.5 Selection of Prediction Methods

We selected the seven methods based on several key criteria: their relevance to cross‐ancestry prediction, demonstrated performance in prior studies, practical aspects like ease of implementation and computational feasibility for large‐scale datasets, and their widespread application in recent years. For each method, we implemented the default function for both modeling and computations to ensure consistency and comparability across the approaches.

### 2.6 GBLUP

Genomic best linear unbiased prediction (GBLUP) is a commonly used statistical method for predicting complex traits using individual‐level data (Yang et al. 2010; VanRaden 2008). This method assumes an identical distribution of SNP effects that are normally distributed with a mean of zero and a variance that is proportional to the genetic variance of the trait being studied (VanRaden 2008; Goddard 2009) (Table 1). It uses a linear mixed model (LMM) to estimate an individual's genetic values based on their genetic similarity to a reference population, as measured by the genomic relationship matrix (GRM) (Lee et al. 2011). GBLUP indirectly accounts for LD through the SNP‐based heritability estimate based on the correlation structure of the genetic effects across SNPs, that is, GRM.

**Table 1:** Summary of the cross‐ancestry genomic prediction methods.

Methods	Data needed	Assumption about the distribution of SNP effects	Modeling and computation	Discovery and target population	Tuning parameter	LD consideration
GBLUP	Individual‐level	$\beta \sim N(0,\frac{h_{g}^{2}}{m})$ $h_{g}^{2}$ is the SNP‐based heritability, m is the number of SNPs	**Modeling:** This method is based on a linear mixed model framework with a genomic relationship matrix (GRM) to capture genetic relatedness between individuals. **Computation**: The method uses matrix algebra to solve for the best linear unbiased predictors of genetic values	It requires genotype and phenotype data from individuals in both discovery and target populations	NA	GBLUP indirectly accounts for LD through the SNP‐based heritability estimate based on the correlation structure of the genetic effects across SNPs, i.e., GRM.
PLINK ‐‐score	Summary statistics	The assumption is similar to GBLUP, but it assumes that the SNPs are in linkage equilibrium.	**Modeling:** This method implements a simple additive model to compute PGS. **Computation:** PGS is computed by summing the products of SNP weights and genotype values for each individual.	It requires GWAS summary statistics as the discovery data set Target samples should have individual level genotype data.	NA	No
PRSice	Summary statistics	The same as PLINK –score approach except it can use an arbitrary p‐value threshold or a range of p‐value thresholds to calculate PGS, allowing LD pruning and clumping of SNPs.	**Modeling:** PRSice allows users to specify thresholds for selecting variants based on their p‐values in the GWAS, enabling fine‐tuning of the score calculation. **Computation:** PRSice is computationally efficient, handling large datasets of genetic variants and phenotypes.	It requires GWAS summary statistics as the discovery data set Target samples should have individual level genotype data.	P‐value threshold	LD pruning and clumping of SNPs.
PRS‐CSx	Summary statistics	$\beta_{\mathrm{jk}}\sim N(0,\frac{\sigma^{2}}{n}\psi_{j})$ *n* is the total number of individuals $\psi_{j}\;\sim \mathrm{Gamma}\;(a,\delta_{j})$ $\delta_{j}\sim \mathrm{Gamma}\;(b,\Phi )$ $\psi_{j}$ is the local shrinkage parameter, Φ is the global shrinkage *a* = 1 and *b* = 1/2 for the Strawderman‐Berger prior ${\hat{\beta }}_{k}=X_{k}^{T}y_{k}/N_{k}$, here the posterior mean of $\beta_{k}$ is $E\;\left[\beta_{k}|\;{\hat{\beta }}_{k}\right]={\left(D_{k}+\psi^{-1}\right)}^{-1}{\hat{\beta }}_{k}$	**Modeling:** Uses a Bayesian framework with continuous shrinkage (CS) priors to estimate posterior effect sizes of genetic variants. **Computation:** Requires GWAS summary statistics and a reference LD panel for each population under analysis. LD matrices are used to model the correlation structure between variants.	GWAS summary statistics from multiple populations can be used as input. PRS‐CSx is particularly effective for target populations with mixed ancestry or those underrepresented in single‐ancestry	$\psi_{j}$ Φ	Population‐specific LD patterns and allele frequencies used in model. LD pattern explicitly modeled in $D_{k}$ matrix
XPASS	Summary statistics + individual‐level	SNP effects follow a bivariate normal distribution, where XPASS estimated the covariance term ($p\sigma_{1}\sigma_{2}$). $\left(\begin{array}{l} \beta_{1,j}\\ \beta_{2,j} \end{array}\right)\sim N$ $\left(\left(\begin{array}{l} 0\\ 0 \end{array}\right),\left(\begin{array}{ll} \sigma_{1}^{2} & p\sigma_{1}\sigma_{2}\\ p\sigma_{1}\sigma_{2} & \sigma_{2}^{2} \end{array}\right)\right)$ Here $\beta_{1,j}$ and $\beta_{2,j}$ are the vector of SNP effect from two populations for SNP *j*. $p\sigma_{1}\sigma_{2}$ is the covariance term which estimated within XPASS	**Modeling:** XPASS integrates GWAS summary statistics from a primary population (source) with LD reference panels from both the primary and secondary populations (target). **Computation:** The method adjusts for differences in LD patterns and allele frequencies between the populations, ensuring that the relationship between effect size and allele frequency.	Requires summary statistics for both discovery and target population. Additionally it requires reference panel for both discovery and target population in plink format.	8 different p‐value thresholds	Considers LD patterns by computing two LD matrices for target and auxiliary population as $R_{1}=X_{1}^{\prime T}X_{1}^{\prime }/m_{1}$ $R_{2}=X_{2}^{\prime T}X_{2}^{\prime }/m_{2}$ For computation of LD matrices, XPASS considers heterogenous pattern of LD between ancestries
BOLT‐LMM	Individual‐level	Slab‐and‐spike mixture of two normal distributions (Zhou et al. 2013) as the prior $\beta_{m}\;\sim N\left(0,\sigma_{\beta ,1}^{2}\right),$ with probability p $\beta_{m}\;\sim N\left(0,\sigma_{\beta ,2}^{2}\right),$ with probability 1 – p If p≪1 and $\sigma_{\beta ,1}^{2}\gg \sigma_{\beta ,2}^{2}$; Slab ($\sigma_{\beta ,1}^{2}$) for assuming small loci with large effects and spike ($\sigma_{\beta ,2}^{2}$) for assuming most SNPs have effects close to zero.	**Modeling:** BoltLMM uses a linear mixed model (LMM) framework to account for population structure and relatedness in GWAS. This helps to reduce false positives caused by cryptic relatedness or population stratification. **Computation:** BoltLMM implements efficient algorithms (e.g., conjugate gradient methods) to handle large‐scale datasets	Single population or mixed populations, with genotype and phenotype data as inputs The target population consists of individuals for whom genetic risk is predicted using PGS derived from BoltLMM GWAS results.	NA	No
PolyPred	Summary stats from BOLT LMM	PolyPred linearly combines SNP effect form BOLT‐LMM and Polyfun Polyfun: $\beta_{i}|a_{i}\approx \left\{\begin{array}{l} N(0,\text{var}\left[\beta_{i}|\beta_{i}\neq 0\right]\;\\ 0\; \end{array}\right.$ $N(0,\text{var}\left[\beta_{i}|\beta_{i}\neq 0\right]$ with prior causal probability $P(\beta_{i}\neq 0|a_{i})$ and var ${[\beta }_{i}|\beta_{i}\neq 0]$ is its causal variance. $a_{i}$ is the functional annotation of SNP *i*. Assuming functional enrichment is primarily due to differences in polygenicity and not to differences in effect size (O'Connor et al. 2019).	**Modeling:** Uses a meta‐analytic approach, combining effect sizes across populations while accounting for population‐specific genetic architecture and LD patterns. **Computation:** It estimates posterior SNP effect sizes by combining GWAS data with LD information, similar to Bayesian approaches (e.g., PRS‐CSx), but it explicitly models cross‐population heterogeneity in effect sizes	Multiple ancestry populations as input as discovery. Polypred is particularly effective in improving PRS performance for target populations with mixed or non‐European ancestries	NA	Using fine‐mapped posterior mean of the causal effect, PolyPred addressed LD differences between ancestries for cross‐ancestry genomic prediction.

### 2.7 PLINK–Score

PLINK–score is a command in the PLINK software package used for scoring individuals based on a set of weights provided in a file of GWAS summary statistics. The weights are used to assign a score to each individual based on their genotype data. This score (i.e., PGS) can then be used to predict phenotypes or assess the individual's genetic risk for a particular disease or trait. It assumes that the effect sizes were calculated using a linear regression model that included the same covariates used in the analysis, and that the variants included in the score were not in LD with each other or other variants not included in the score (Table 1).

### 2.8 PRS‐CSx

PRS‐CSx is a statistical method used to estimate PGS for complex traits using summary statistics from GWAS. It is an extension of the original PRS‐CS method that incorporates external reference panels to improve the accuracy of the PGS estimation. The PRS‐CSx method estimates the effect sizes of SNPs based on LD patterns within the reference panel only during the model training step and not in the validation or testing phases, and these effect sizes are then used to calculate a weighted sum of an individual's genotypes at each SNP. This results in a PGS that reflects the individual's genetic risk for the trait of interest. By introducing continuous shrinkage prior, this method also uses population specific LD patterns and allele frequencies (SNPs effect sizes vary across populations). The effect size of the SNP in the population is modeled as a global–local scale mixture of normal distributions (Table 1).

### 2.9 PRSice

PRSice is a software tool used for calculating and visualizing PGS from GWAS data. It provides a user‐friendly interface for selecting and filtering SNPs based on *p*‐value thresholds ($p_{T}$), LD, and other parameters. PRSice allows for customization of the PGS calculation, including the choice of effect sizes, SNP weighting schemes, and thresholding methods. Additionally, it can perform cross‐validation and generate various visualizations, including PGS distributions plots (Table 1).

### 2.10 XPASS

This method constructs PGS using both individual‐level (XPA) and summary‐level (XPASS) GWAS data. XPA takes individual‐level genetic data as input and offers logical estimates for the SNP effect sizes by leveraging participant information across populations. It can efficiently calculate and accurately construct risk scores in target ancestries using algorithms such as Boolean representation (Wan et al. 2010; Loh et al. 2015) and stochastic approximation (Wu and Sankararaman 2018). To improve risk prediction in non‐European populations, XPA leverages the trans‐ancestry genetic correlation of the trait between populations due to the shared genetic basis. XPASS, on the other hand, considers heterogeneous patterns of LD between the target and discovery ancestries. To further improve PGS construction, it also incorporates population‐specific effects. XPA introduces probability structures for $\beta_{1}$ and $\beta_{2}$ to model polygenic effects and the correlation between ancestries (Table 1).

### 2.11 BOLT‐LMM

Bayesian and Lasso‐based Oligogenic Linkage Analysis with Refined Treatment (BOLT‐LMM) is a software package used for performing genome‐wide association studies (GWAS) and heritability analyzes. BOLT‐LMM uses a mixed linear model (MLM) approach to account for population structure and relatedness among individuals in GWAS. This approach allows for a more accurate estimation of the genetic effects of variants on complex traits. Additionally, BOLT‐LMM incorporates a Bayesian approach to identify oligogenic genetic effects (i.e., the effects of multiple genetic variants acting in combination) and a Lasso‐based approach to select the most informative variants (Table 1). This model assumes that the SNP effects were calculated following Slab‐and‐spike mixture (a type of Bayesian linear regression) of two normal distributions (Zhou et al. 2013).

### 2.12 PolyPred

PolyPred is a method that improves the accuracy of PGS by combining two approaches: BOLT‐LMM (Loh et al. 2015) and PolyFun (Weissbrod et al. 2020). BOLT‐LMM estimates the effect sizes of SNPs, while PolyFun uses fine mapping techniques such as SuSIE (Wang et al. 2020b) and FINEMAP (Benner 2016, 2025) to estimate the association effects of SNPs based on inferred priors. By combining the results from these two methods, PolyPred produces a more accurate and robust estimation of PGS than using each approach individually. Typically, PolyPred is trained on a European discovery dataset (Table 1).

### 2.13 Evaluation of Cross Ancestry Prediction Methods

We evaluated the effectiveness of cross‐ancestry genomic prediction methods using real data from the UK Biobank, which encompasses both highly polygenic traits, such as BMI and standing height, as well as less polygenic traits, like HDL‐ and LDL‐cholesterol. We compared the predictive ability ($R^{2}$) of PGS across ancestries using seven existing prediction methods. To assess and quantify the differences in performance across methods and within target ancestries, we utilized the r2redux (Momin et al. 2023b) R‐package. This package estimates the variance and covariance of values, providing a 95% confidence interval (CI) and p‐value for the difference in $R^{2}$ between two sets of dependent or independent PGS. By comparing the performance across methods, we were able to identify the best‐performing method(s) for predicting traits across ancestries. If the performance of any method did not show a significant difference compared to the best method, we considered it as one of the best performing methods.

### 2.14 Rationale of Comparing Cross‐Ancestry Prediction Versus Ancestry Matched Prediction

In genomic prediction, aligning training and test sets from the same population is typically prioritized for optimizing prediction accuracy. However, our comparison of cross‐ancestry and ancestry‐matched prediction approaches addresses unique challenges specific to polygenic trait prediction, especially given the limited diversity in current training data. This study highlights key differences between these approaches for several reasons. First, while ideally, prediction models utilize training data from the same population as the test set, this is often impractical in polygenic prediction due to the disproportionate representation of European populations in most datasets. Our study showcases the impact of this imbalance, particularly on non‐European populations. Additionally, cross‐ancestry prediction brings critical challenges in genetics where ancestry‐specific genetic architecture greatly influences trait prediction accuracy, and our analysis quantifies how model performance deteriorates when applied across diverse populations. This underscores the need for methods that address these ancestry‐specific gaps. By comparing traditional ancestry‐matched models with those designed for cross‐ancestry applications, we demonstrate the effectiveness of newer, inclusive methods that improve prediction accuracy across populations, highlighting areas for future model enhancement. While ideally, training data would align with target populations, the current reality necessitates effective cross‐ancestry predictions to address data limitations, and our study evaluates methods that address these real‐world challenges. Ultimately, our work advocates for greater inclusivity in genomic prediction by emphasizing the importance of reliable methods across varied populations, not only as a technical improvement but as an ethical imperative to ensure genomic advancements benefit diverse groups equitably.

## 3 Results

### 3.1 Cross‐Ancestry Prediction Accuracy Across Methods

We compared the performance of seven methods in cross‐ancestry prediction, which include four summary statistics‐based methods (PLINK, PolyPred, PRS‐CSx, and PRSice) and three individual‐level data‐based methods (BOLT‐LMM, GBLUP and XPASS). To assess the prediction performance across the methods, we used $R^{2}$ that is a well‐established metric for the polygenic score models, and its comparative significance test was carried out by an R‐package, *r2redux (*Momin et al. 2023b *)* (see Methods). The P‐value for the significant difference between each pair of the methods for the traits and ancestries is shown in Supporting Information Tables S3–S7. In the cross‐ancestry prediction, we used 50,000 or 258,792 White British individuals as the discovery dataset to predict unobserved phenotypes of other ancestry groups such as Other Europeans (*n* = 26,457), South Asians (6199) and Africans (6179) as the target datasets.

**Figure 1:**
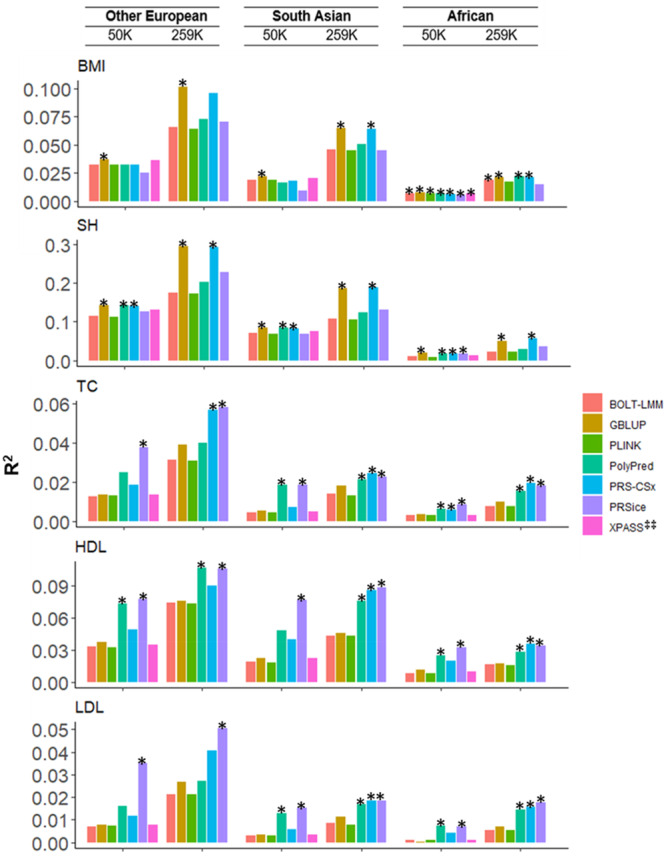
Comparison of cross‐ancestry prediction methods: Evaluation based on $R^{2}$ values and comparative significance testing to determine the best‐performing methods. BMI, body mass index; HDL, high‐density lipoprotein cholesterol; LDL, low‐density lipoprotein cholesterol; SH, standing height; TC, total cholesterol. The predictive ability ($R^{2}$) of cross‐ancestry polygenic risk scores depends on the polygenicity of the trait and the sample size of discovery dataset. BMI and SH are known to be highly polygenic, and cholesterol traits are less polygenic (Snieder et al. 1999). PGS were constructed based on SNP effects estimated using 50,000 or 258,792 White British individuals as the discovery dataset. We used Other European, South Asian, and African as the target datasets (*n* = 26,457, 6199, and 6179, respectively) that are independent from the White British discovery dataset. For other Europeans, South Asians, and Africans, we used SNP # 1,138,117, 904,421, and 632,906, respectively, which are common with white British. The bars represent $R^{2}$ values of polygenic risk scores evaluated in the target dataset. Asterisk signs on the top of the bars indicate that the $R^{2}$ values are not significantly different from the highest $R^{2}$ (i.e., the best methods). P‐values of the comparative significance test to assess difference between methods are shown in Supporting Information Tables S1–S5, which were estimated using R‐package *r2redux (*Momin et al. 2023b *)*. ^‡‡^XPASS was not used in the case of 258,792 white British discovery because of its high computational demands.

As expected, increasing the discovery sample size improved cross‐ancestry prediction (Figure 1). For highly polygenic traits like BMI and standing height, GBLUP and PRS‐CSx outperformed other methods in predicting Other European and South Asian ancestries (Figure 1). When considering African ancestry, there was a lack of power to identify the best method although GBLUP and PRS‐CSx were always in the best group (asterisk) whether using BMI or standing height (Figure 1). For less polygenic traits like cholesterol, PRSice and PolyPred were usually the best methods with a sample size of 50,000 (Figure 1). When the sample size was increased to 258,792, PRS‐CSx demonstrated improved performance relative to other methods (Supporting Information Figure S1) and was often among the top performers, along with PRSice and PolyPred (Figure 1). Overall, PRSice is the best method with less polygenic traits (Figure 1 and Supporting Information Figures S2 and S3).

### 3.2 Cross‐Ancestry Prediction Versus Ancestry‐Matched Prediction

The accuracy of PGS predictions is influenced by various genetic factors, including the polygenicity, heritability and genetic correlation of the trait between the discovery and target populations. As demonstrated in Figure 1, PGS generated from European GWAS summary statistics tend to have reduced predictive accuracy when applied to non‐European populations for all traits. The differences in accuracy between cross‐ancestry and ancestry‐matched predictions varied across traits. Here, cross‐ancestry prediction refers to using PGS generated from White British GWAS summary statistics to predict non‐European samples, while ancestry‐matched prediction involves predicting Other European samples. It is worth noting that White British and Other European populations are generally considered to be ancestry‐matched due to their genetic similarity although there can possibly be some degree of genetic variation between these populations.

**Figure 2:**
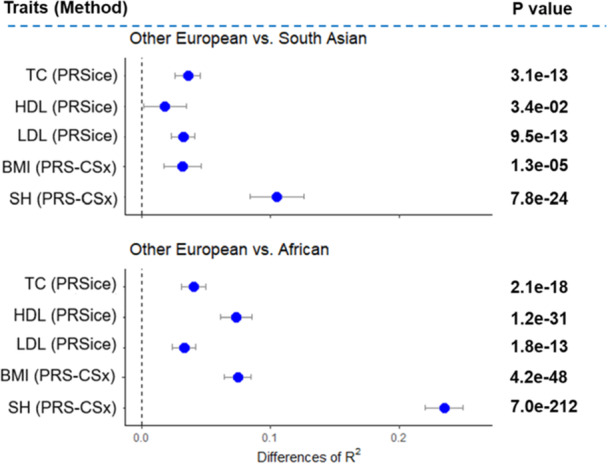
The difference of predictive ability ($R^{2}$) of polygenic risk scores between within‐European prediction versus cross‐ancestry prediction. BMI, body mass index; HDL, high‐density lipoprotein cholesterol; LDL, low‐density lipoprotein cholesterol; SH, standing height; TC, total cholesterol. The best‐performing method was used for each ancestry and trait, as shown in Figure 1. We estimated SNP effects using a discovery dataset of 258,792 individuals of White British ancestry. The dot points represent the differences in $R^{2}$ between cross‐ancestry prediction and within‐European prediction (difference = within‐European prediction $R^{2}$ – cross‐ancestry prediction $R^{2}$), and the error bars indicate the 95% confidence intervals of the difference. A positive difference value indicates that within‐European prediction performs better than cross‐ancestry prediction. The p‐values in the figure indicate that the differences in $R^{2}$ are significantly different from zero and were estimated using R‐package *r2redux* (Momin et al. 2023b).

When comparing ancestry‐matched and cross‐ancestry predictions (e.g., predicting Other European vs. South Asian ancestry), we observed greater differences in accuracy for highly polygenic and heritable traits such as height (as illustrated in Figure 2). However, for less polygenic traits such as cholesterol, the difference between ancestry‐matched and cross‐ancestry predictions reduced toward zero, particularly for HDL‐cholesterol in South Asian populations (shown in Figure 2). Similarly, when comparing Other European and African ancestries, highly polygenic and heritable traits showed greater differences in accuracy between ancestry‐matched and cross‐ancestry predictions, while less polygenic traits exhibited smaller differences (as illustrated in Figure 2).

### 3.3 Cross‐Ancestry Prediction Using SNPs Having Concordant Effects Between Two Ancestries

**Figure 3:**
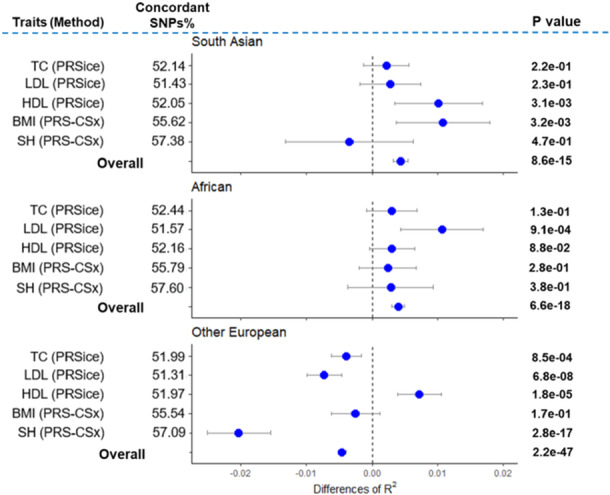
The difference of predictive ability ($R^{2}$) of polygenic risk scores between concordant SNPs and total SNPs for five complex traits. BMI, body mass index; HDL, high‐density lipoprotein cholesterol; LDL, low‐density lipoprotein cholesterol; SH, standing height; TC, total cholesterol. For each ancestry per trait, the best‐performing method was used (see Figure 1). The SNP effects were estimated using a discovery dataset consisting of 258,792 individuals of White British ancestry. Concordant SNPs were identified by comparing the direction of SNP effects between two diverse populations (the GWAS of white British from UK Biobank and the GWAS from Biobank Japan). Dot points represent the differences in $R^{2}$ values (i.e., difference = concordant SNP $R^{2}$ – total SNP $R^{2}$), while error bars indicate 95% confidence intervals of the difference. A difference value greater than zero indicates that the concordant SNPs perform better than the total SNPs, even though the number of concordant SNPs is only around 50% of the total SNPs. The overall $R^{2}$ value is obtained from a meta‐analysis of five traits. The p‐values in the figure indicate that the differences in $R^{2}$ values are significantly different from zero, which were estimated using R‐package *r2redux* (Momin et al. 2023b).

It has been reported that some genes are functionally homogeneous across ancestries (Peterson et al. 2019; Lam et al. 2019; Marigorta and Navarro 2013). Comparing the direction of SNP effects between two diverse ancestries can help identify SNPs associated with functionally homogeneous genes (Stranger et al. 2007). To identify the set of concordant SNPs for each trait, we compared two sets of GWAS summary statistics: one from the Biobank Japan (BBJ) dataset (n = 70k – 158k) and the other from a dataset of 258,792 individuals with white British ancestry. Neither of these datasets overlapped with our target individuals, who were drawn from other European, South Asian, and African ancestry groups. We then tested whether using this set of SNPs could improve the performance of cross‐ancestry predictions for each trait (Figure 3). Our results showed that the overall improvement in terms of $R^{2}$ was 0.0043 (95% CI 0.0032–0.0054, *p*‐value 8.62e‐15) for South Asian and 0.0039 (95% CI 0.0030–0.0049, *p*‐value 6.63e‐18) for African ancestry when using concordant SNPs. However, for Other European ancestry, the overall improvement was ‐0.0045 (95% CI ‐0.0052 to ‐0.0039, *p*‐value 2.21e‐47), indicating that using all SNPs performs better than using concordant SNPs only (Figure 3, Supporting Information Figures S4–S6). This suggests that the concordant SNPs are significantly more useful than the total set of SNPs when using cross‐ancestry predictions. However, the remaining SNPs without the concordant set might still be useful in predicting the same European ancestry, even though they may not contribute to predicting other non‐European ancestries.

## 4 Discussion

Cross‐ancestry polygenic prediction shows promise in addressing genomic health disparities caused by the underrepresentation of non‐European ancestries in genomic databases. Therefore, it is critical to evaluate the effectiveness of current cross‐ancestry prediction methods for a range of traits, including highly polygenic traits such as BMI and standing height, and less polygenic traits such as cholesterol. In our study, we observed that GBLUP and PRS‐CSx demonstrated superior performance in predicting highly polygenic traits such as height and BMI, compared to other methods. On the other hand, PRSice and PolyPred outperformed other methods for less polygenic traits like cholesterol. Notably, PRS‐CSx also showed comparable performance with larger sample sizes, indicating its potential as a reliable method for polygenic prediction in cross‐ancestry studies. We have also identified the importance of incorporating concordant SNPs, which have the same effect direction across diverse ancestries, to enhance the accuracy of cross‐ancestry PGS models. Additionally, our study demonstrated that the transferability of PGS across ancestries is trait‐specific, highlighting the necessity for more investigation in this field.

Our analysis revealed that achieving accurate predictions in non‐European populations using PGS based on European discovery individuals remains a significant challenge due to differences in allele frequencies (Martin et al. 2019; Wang et al. 2020a; Durvasula and Lohmueller 2021; Graham et al. 2021), LD patterns (Martin et al. 2019; Wang et al. 2020a; Graham et al. 2021), and environmental exposures between populations (Zhou and Lee 2021). However, we also found that the accuracy of cross‐ancestry prediction varies depending on the trait being predicted. Interestingly, our analysis suggests that cross‐ancestry prediction accuracy may be relatively more preserved for less polygenic traits such as cholesterol, although this interpretation should be made cautiously given the differences in baseline prediction accuracy across traits. On the other hand, for highly polygenic and heritable traits such as height, the difference in accuracy between cross‐ancestry and ancestry‐matched predictions is more pronounced. These findings have important implications for genomic medicine, particularly in the context of using European discovery samples to predict non‐European populations. To improve the accuracy of predictions for diverse populations, it is therefore crucial to carefully consider the choice of prediction method and the availability of relevant reference data, given the trait‐specific differences in accuracy. Our study suggests that a more tailored approach is necessary to accurately predict highly polygenic and heritable traits in non‐European populations.

We conducted a comparison of the direction of SNP effect between UK Biobank and Biobank Japan GWAS summary statistics to identify concordant SNP sets (Momin et al. 2024). Importantly, the two datasets used in this analysis are independent of the three target ancestry groups that were used in our study. Our study demonstrates the importance of using concordant SNPs in cross‐ancestry polygenic prediction. We found that the prediction accuracy of concordant SNPs was significantly better than that of the total set of SNPs (Momin et al. 2024), likely because they are more likely to be causal variants or in LD with causal variants across ancestries. Previous studies have shown that concordant SNPs are typically enriched in regulatory and genic regions (Momin et al. 2024). It is worth noting that concordant SNPs did not perform better than the remaining SNPs in ancestry‐matched prediction, likely because discordant SNPs are still in LD with causal SNPs in the ancestry‐matched populations. Another study reported an improvement in cross‐ancestry prediction accuracy by using cell‐type‐specific regulatory elements that are consistent across European and East Asian populations to predict gene expression levels, that is, concordant transcriptomic effects (Amariuta et al. 2019, 2020). Although this study focused on the transcriptome rather than the genome, its implications for cross‐ancestry prediction are similar to ours. Therefore, a further study that combines information from both the genome and transcriptome may be warranted to improve cross‐ancestry polygenic prediction accuracy.

This study has several limitations that should be noted. Firstly, the cross‐ancestry genomic prediction analysis was restricted to HapMap3 SNPs and common variants with a minor allele frequency (MAF) greater than 0.01. This may have excluded potentially informative variants that are rare in the study populations. Secondly, the identification of concordant SNPs was based on a comparison of SNP effects between the white British (UKBB) and East Asian (BBJ) populations due to the lack of adequate data from other ancestries. While these populations were chosen for their large sample sizes, the concordant SNP sets identified may not generalize to other populations. Thirdly, to compare the methods, we utilized their default parameter settings under the assumption that these settings are generally robust. However, it is important to note that prediction performance may be further optimized by adjusting parameters in specific cases. Therefore, future studies could explore the impact of parameter tuning on prediction accuracy for each method. Fourthly, this study did not include all available PGS methods as some methods require additional information. For instance, LDpred2 was excluded from this analysis because a previous study (Ruan et al. 2022) demonstrated that PRS‐CSx outperformed LDpred2 in cross‐ancestry prediction. Additionally, PolyPred+ and PolyPred‐S+ were not evaluated in this study because they require additional discovery samples from ancestry‐matched targets. To ensure a fair comparison across methods, this study focused exclusively on those that did not require additional samples. While this approach may have limited the scope of the analysis, it allowed for a more straightforward comparison of the methods under similar conditions. Nonetheless, future studies should consider incorporating a wider range of PGS methods to better understand their comparative performance. Finally, we did not compare methods that use discovery samples from multiple ancestries to predict phenotypes in target samples, as our focus was on the realistic scenario of predicting non‐European target samples using European discovery samples.

In conclusion, our findings suggest that the choice of prediction method should be tailored to the trait being analyzed, the type of data available, and the population of interest. For highly polygenic traits such as, BMI and standing height, we recommend using GBLUP for individual‐level data and PRS‐CSx for summary‐based data to achieve optimal prediction performance. For less polygenic traits like cholesterol, PRSice consistently performed well in all comparisons. Furthermore, we recommend using concordant SNPs instead of the total SNP set for cross‐ancestry prediction (https://www.ebi.ac.uk/gwas/; details are in web resources section). Notably, a more tailored approach is required to accurately predict highly polygenic and heritable traits like height in non‐European populations when using European discovery samples. However, it is important to note that these recommendations may not be universally applicable, and specific adjustments to the method parameters may be necessary in certain cases to optimize prediction accuracy.

## 1 Web Resources and Code Availability

The genotype and phenotype data of the UK Biobank can be accessed through procedures described on its webpage (https://www.ukbiobank.ac.uk/) and summary statistics of BMI, total‐, HDL‐, and LDL‐cholesterol of Japanese Biobank (BBJ) can be obtained from their website (http://jenger.riken.jp/en/).

MTG2 version can be downloaded from (https://sites.google.com/view/s-hong-lee-homepage/mtg2).

PLINK version can be downloaded from (https://www.cog-genomics.org/plink2/).

PRSice version can be downloaded from (https://choishingwan.github.io/PRS-Tutorial/prsice/).

PRS‐CSx version can be downloaded from (https://github.com/getian107/PRScsx).

XPASS version can be downloaded from (https://github.com/YangLabHKUST/XPASS).

PolyPred versions can be downloaded from (https://github.com/omerwe/polyfun).

BOLT‐LMM versions can be downloaded from (http://www.hsph.harvard.edu/alkes-price/software/).

r2redux can be downloaded from (https://github.com/mommy003/r2redux or from CRAN.

Example codes are available on GitHub (https://github.com/mommy003/pred_codes/).

GWAS of BMI (all SNP)

https://ftp.ebi.ac.uk/pub/databases/gwas/summary_statistics/GCST90268001-GCST90269000/GCST90268031/

GWAS of BMI (concordant SNP)

https://ftp.ebi.ac.uk/pub/databases/gwas/summary_statistics/GCST90268001-GCST90269000/GCST90268032/

GWAS of Standing Height (all SNP)

https://ftp.ebi.ac.uk/pub/databases/gwas/summary_statistics/GCST90268001-GCST90269000/GCST90268033/

GWAS of Standing Height (concordant SNP)

https://ftp.ebi.ac.uk/pub/databases/gwas/summary_statistics/GCST90268001-GCST90269000/GCST90268034/

GWAS of total cholesterol (all SNP) (https://ftp.ebi.ac.uk/pub/databases/gwas/summary_statistics/GCST90244001-GCST90245000/GCST90244051/)

GWAS of total cholesterol (concordant SNP) (https://ftp.ebi.ac.uk/pub/databases/gwas/summary_statistics/GCST90244001-GCST90245000/GCST90244052/)

GWAS of HDL‐cholesterol (all SNP) (https://ftp.ebi.ac.uk/pub/databases/gwas/summary_statistics/GCST90244001-GCST90245000/GCST90244053/)

GWAS for HDL‐cholesterol (concordant SNP) (https://ftp.ebi.ac.uk/pub/databases/gwas/summary_statistics/GCST90244001-GCST90245000/GCST90244054/)

GWAS for LDL‐cholesterol (all SNP) (https://ftp.ebi.ac.uk/pub/databases/gwas/summary_statistics/GCST90244001-GCST90245000/GCST90244055/)

GWAS for LDL‐cholesterol (concordant SNP) (https://ftp.ebi.ac.uk/pub/databases/gwas/summary_statistics/GCST90244001-GCST90245000/GCST90244056/)

## Conflicts of Interest

The authors declare no conflicts of interest.

## Supporting information


**Figure S1:** The comparison of predictive ability of total cholesterol between PRS‐CSx and PRSice across different discovery sample sizes. **Figure S2:** The comparison of predictive ability of HDL‐cholesterol between PRS‐CSx and PRSice across different discovery sample sizes. **Figure S3:** The comparison of predictive ability of LDL‐cholesterol between PRS‐CSx and PRSice across different discovery sample sizes. **Figure S4:** The difference of predictive ability () of polygenic risk scores between concordant SNPs and total SNPs for four complex traits across three methods in South Asian. **Figure S5:** The difference of predictive ability () of polygenic risk scores between concordant SNPs and total SNPs for four complex traits across three methods in African. **Figure S6:** The difference of predictive ability () of polygenic risk scores between concordant SNPs and total SNPs for four complex traits across three methods in Other European. **Table S1:** The total number and percentage of common concordant and discordant when comparing SNP effect between UK Biobank and Biobank Japan. **Table S2:** The number and percentage of common concordant and discordant SNPs from HapMap3 SNPs for each pair of ancestries across traits. **Table S3:** P‐value for the significant difference between each pair of the methods across ancestries for BMI while using 50,000 white British as discovery. **Table S4:** P‐value for the significant difference between each pair of the methods across ancestries for standing height while using 50,000 white British as discovery. **Table S5:** P‐value for the significant difference between each pair of the methods across ancestries for total cholesterol while using 50,000 white British as discovery. **Table S6:** P values to test the significance of difference between methods across ancestries for HDL‐cholesterol while using 50,000 white British as discovery. **Table S7:** P values to test the significance of difference between methods across ancestries for LDL‐cholesterol while using 50,000 white British as discovery.
