# Supplementary material for: Cross‐Ancestry Polygenic Prediction: Comparing Methods and Assessing Transferability Across Traits
Source: Genet Epidemiol. 2026 Jan 21;50(1):e70029. doi: 10.1002/gepi.70029 (PMC12820924; doi:10.1002/gepi.70029)
Supplement: Supplementary file 1 — Figure S1: The comparison of predictive ability of total cholesterol between PRS‐CSx and PRSice across different discovery sample sizes. Figure S2: The comparison of predictive ability of HDL‐cholesterol between PRS‐CSx and PRSice across different discovery sample sizes. Figure S3: The comparison of predictive ability of LDL‐cholesterol between PRS‐CSx and PRSice across different discovery sample sizes. Figure S4: The difference of predictive ability () of polygenic risk scores between concordant SNPs and total SNPs for four complex traits across three methods in South Asian. Figure S5: The difference of predictive ability () of polygenic risk scores between concordant SNPs and total SNPs for four complex traits across three methods in African. Figure S6: The difference of predictive ability () of polygenic risk scores between concordant SNPs and total SNPs for four complex traits across three methods in Other European. Table S1: The total number and percentage of common concordant and discordant when comparing SNP effect between UK Biobank and Biobank Japan. Table S2: The number and percentage of common concordant and discordant SNPs from HapMap3 SNPs for each pair of ancestries across traits. Table S3: P‐value for the significant difference between each pair of the methods across ancestries for BMI while using 50,000 white British as discovery. Table S4: P‐value for the significant difference between each pair of the methods across ancestries for standing height while using 50,000 white British as discovery. Table S5: P‐value for the significant difference between each pair of the methods across ancestries for total cholesterol while using 50,000 white British as discovery. Table S6: P values to test the significance of difference between methods across ancestries for HDL‐cholesterol while using 50,000 white British as discovery. Table S7: P values to test the significance of difference between methods across ancestries for LDL‐cholesterol while using 50,000 white [file GEPI-50-0-s001.docx]

**Supplementary Information’s**

**
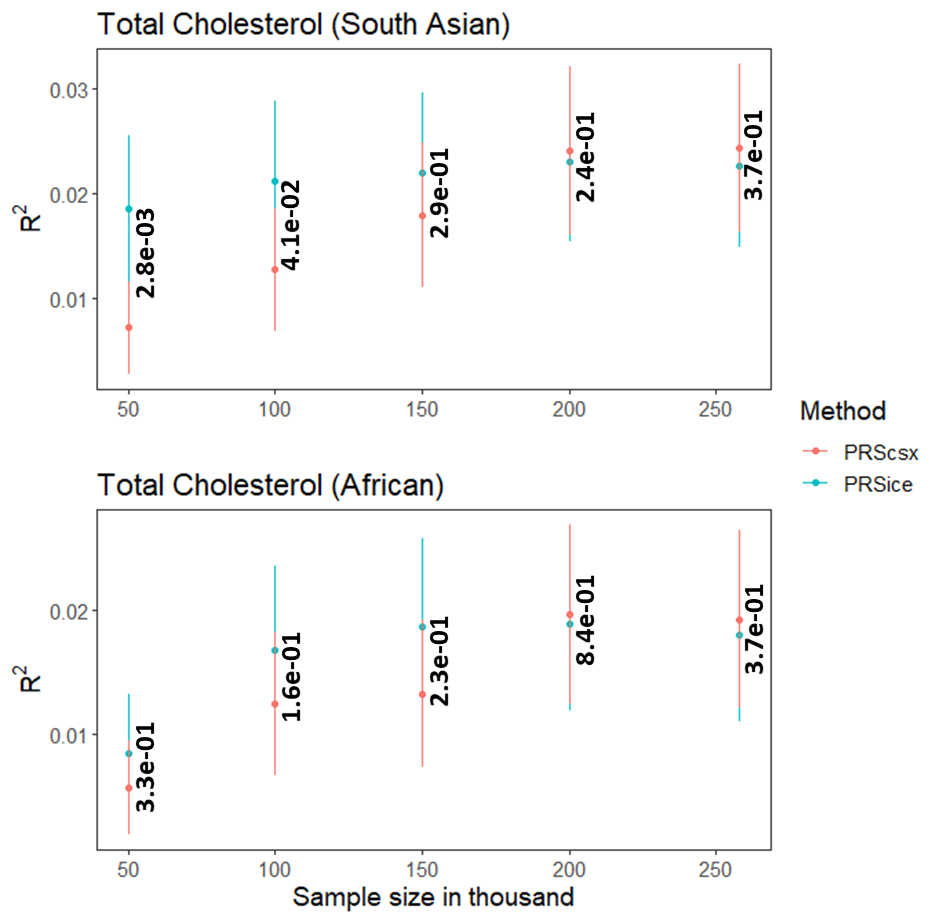
**

**Figure S1: The comparison of predictive ability of total cholesterol between PRS-CSx and PRSice across different discovery sample sizes.** Dot points represent the $R^{2}$ values for PRScsx and PRSice across different sample sizes and error bars indicate 95% confidence intervals. Numeric values are the P-values to test the significance of difference between methods (PRS-CSx and PRSice). P-values was estimated using an R-package (r2redux)^1^.

**
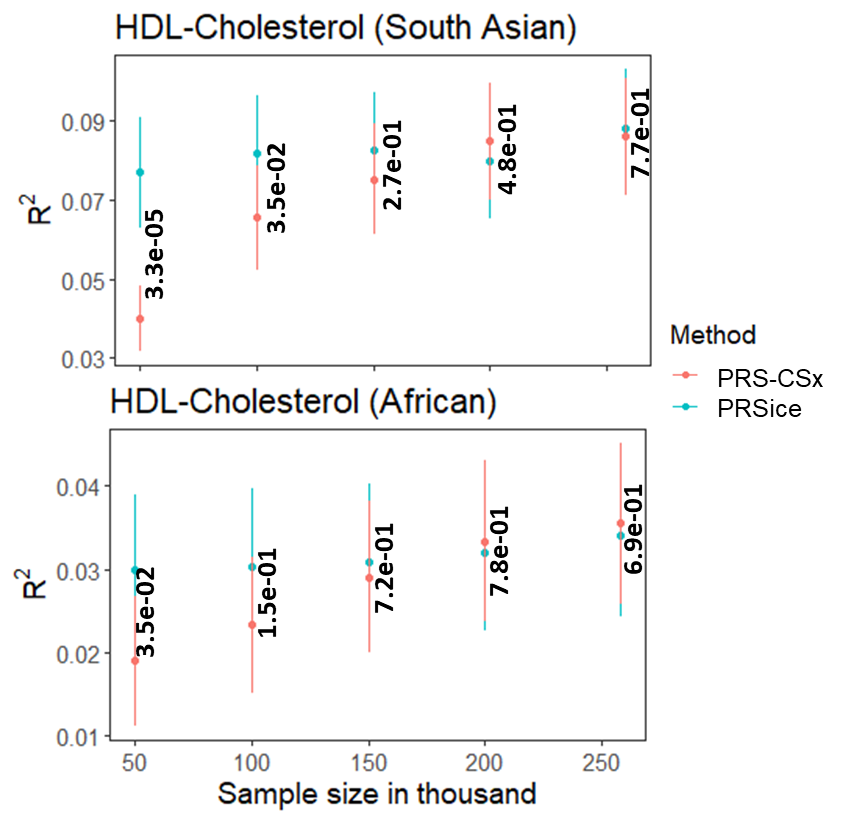
**

**Figure S2: The comparison of predictive ability of HDL-cholesterol between PRS-CSx and PRSice across different discovery sample sizes.** Dot points represent the $R^{2}$ values for PRScsx and PRSice across different sample sizes and error bars indicate 95% confidence intervals. Numeric values are the P-values to test the significance of difference between methods (PRS-CSx and PRSice). P-values was estimated using an R-package (r2redux)^1^.

**
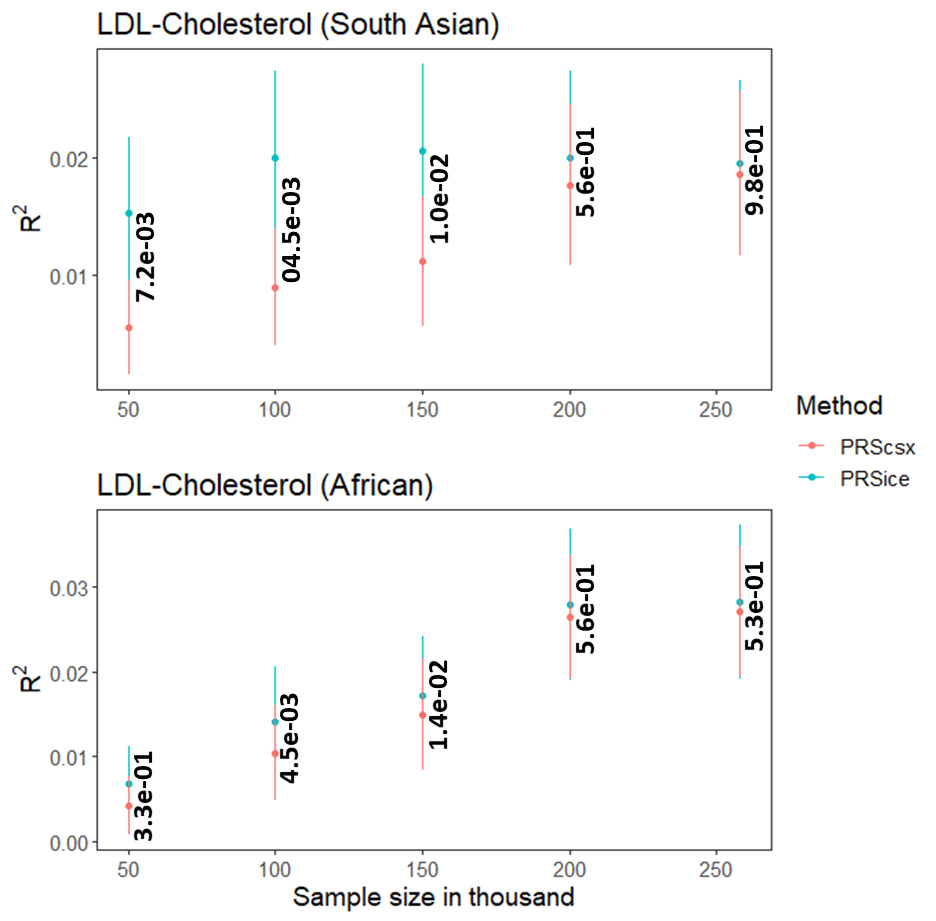
**

**Figure S3: The comparison of predictive ability of LDL-cholesterol between PRS-CSx and PRSice across different discovery sample sizes.** Dot points represent the $R^{2}$ values for PRScsx and PRSice across different sample sizes and error bars indicate 95% confidence intervals. Numeric values are the P-values to test the significance of difference between methods (PRS-CSx and PRSice). P-values was estimated using an R-package (r2redux)^1^.

**
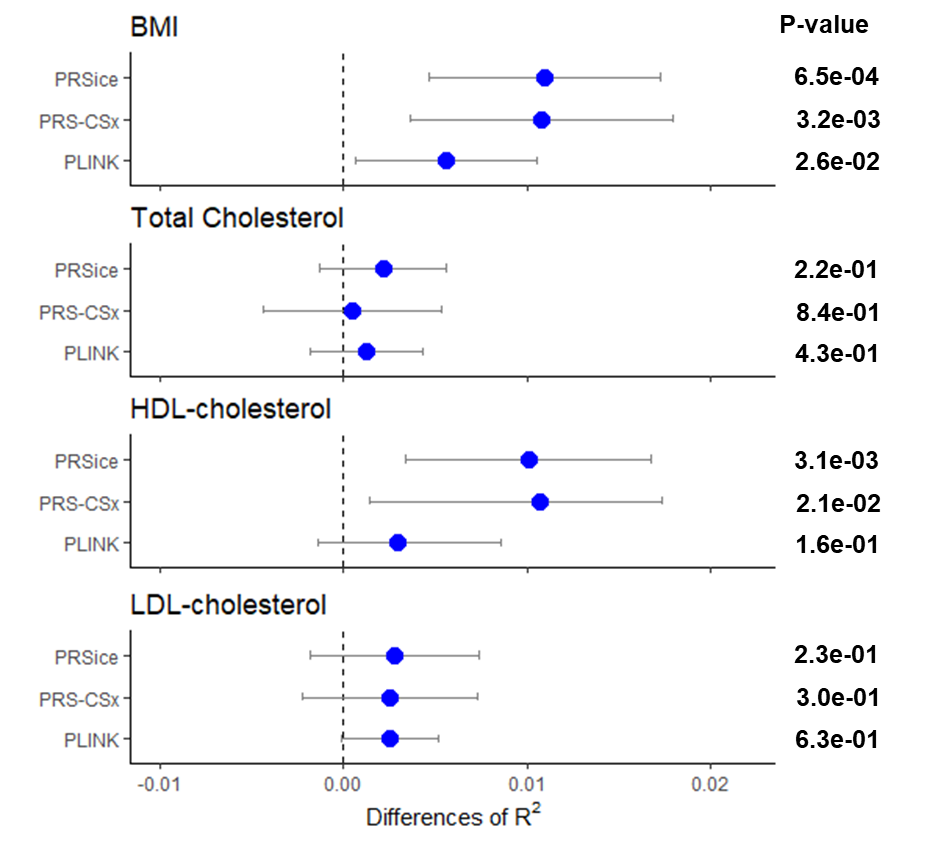
**

**Figure S4:** **The difference of predictive ability (**$\boldsymbol{R}^{\boldsymbol{2}}$**) of polygenic risk scores between concordant SNPs and total SNPs for four complex traits across three methods in South Asian.** SNP effects were estimated using ~258k White British ancestry cohort as the discovery dataset. Dot points represent the differences of $R^{2}$ (difference = concordant SNP $R^{2}$ – total SNP $R^{2}$), and error bars indicate 95% confidence intervals of the difference. The difference > 0 means that the concordant SNPs perform better than the total SNPs although the number of concordant SNPs is around 50% of the total SNPs. The *p*-values indicate that the differences of $R^{2}$ are significantly different from zero. P-values was estimated using an R-package (r2redux)^1^. Here, we do not include result for standing height because of an insufficient number of SNPs matched between UK Biobank and Biobank Japan for the trait.


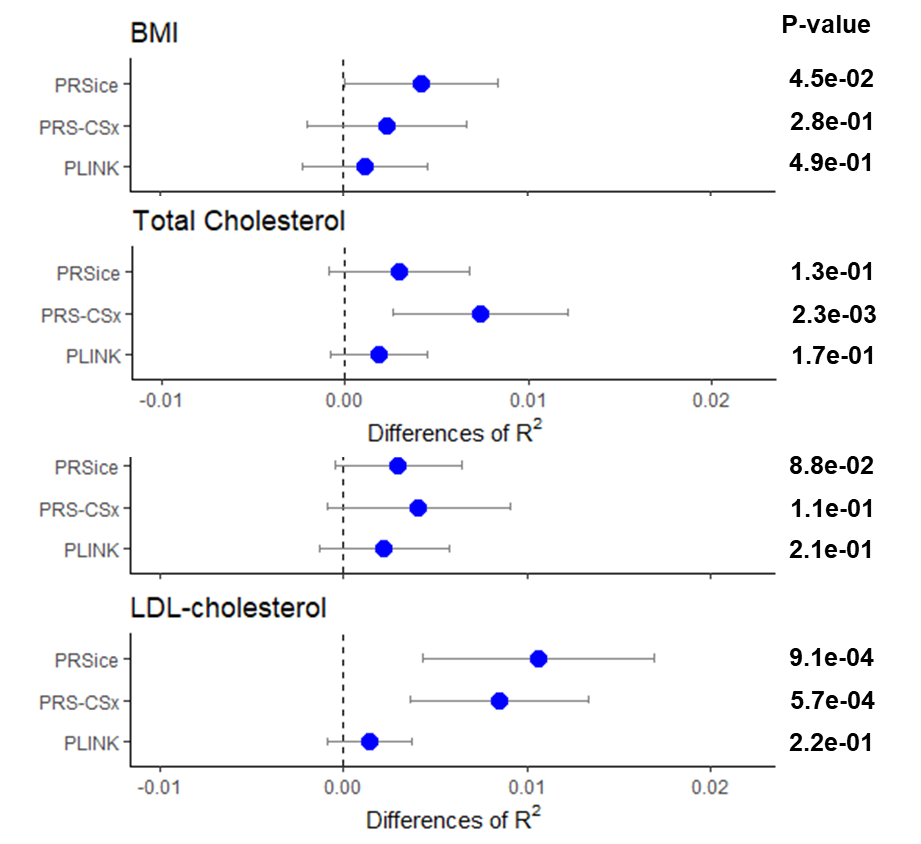


**Figure S5:** **The difference of predictive ability (**$\boldsymbol{R}^{\boldsymbol{2}}$**) of polygenic risk scores between concordant SNPs and total SNPs for four complex traits across three methods in African.** SNP effects were estimated using ~258k White British ancestry cohort as the discovery dataset. Dot points represent the differences of $R^{2}$ (difference = concordant SNP $R^{2}$ – total SNP $R^{2}$), and error bars indicate 95% confidence intervals of the difference. The difference > 0 means that the concordant SNPs perform better than the total SNPs although the number of concordant SNPs is around 50% of the total SNPs. The *p*-values indicate that the differences of $R^{2}$ are significantly different from zero. P-values was estimated using an R-package (r2redux)^1^. Here, we do not include result for standing height because of an insufficient number of SNPs matched between UK Biobank and Biobank Japan for the trait.


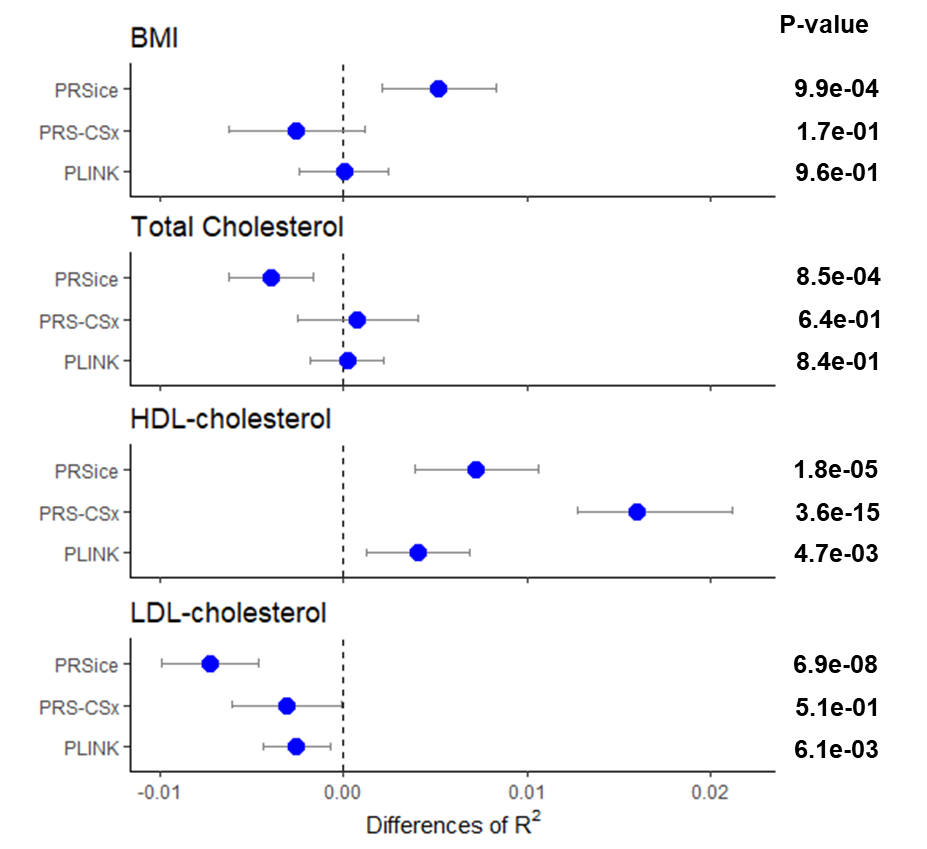


**Figure S6:** **The difference of predictive ability (**$\boldsymbol{R}^{\boldsymbol{2}}$**) of polygenic risk scores between concordant SNPs and total SNPs for four complex traits across three methods in Other European.** SNP effects were estimated using ~258k White British ancestry cohort as the discovery dataset. Dot points represent the differences of $R^{2}$ (difference = concordant SNP $R^{2}$ – total SNP $R^{2}$), and error bars indicate 95% confidence intervals of the difference. The difference > 0 means that the concordant SNPs perform better than the total SNPs although the number of concordant SNPs is around 50% of the total SNPs. The *p*-values indicate that the differences of $R^{2}$ are significantly different from zero. P-values was estimated using an R-package (r2redux)^1^. Here, we do not include result for standing height because of an insufficient number of SNPs matched between UK Biobank and Biobank Japan for the trait.

**Table S1:** The total number and percentage of common concordant and discordant when comparing SNP effect between UK Biobank and Biobank Japan.

| **Traits** | **Concordant SNPs** | **Discordant SNPs** |
| --- | --- | --- |
| **Standing Height** | 1,963,734 (57.05%) | 1,478,338 (42.95%) |
| **BMI** | 2,280,806 (55.45%) | 1,832,824 (44.55%) |
| **Total-cholesterol** | 2,139,361 (52.01%) | 1,974,269 (47.99%) |
| **HDL-cholesterol** | 2,133,129 (51.86%) | 1,980,501 (48.14%) |
| **LDL-cholesterol** | 2,105,165 (51.18%) | 2,008,465 (48.82%) |

**Table S2:** The number and percentage of common concordant and discordant SNPs from HapMap3 SNPs for each pair of ancestries across traits.

| **Traits** | **Ancestries** | **No of concordant SNPs** | **No of discordant SNPs** |
| --- | --- | --- | --- |
| **Standing Height** | White British vs. Other European | 454,453 (57.09%) | 341,521 (42.91%) |
|  | White British vs. South Asian | 357,285 (57.38%) | 265,349 (42.62%) |
|  | White British vs. African | 252,844 (57.60%) | 186,136 (42.40%) |
| **BMI** | White British vs. Other European | 494,306 (55.54%) | 395,726 (44.44%) |
|  | White British vs. South Asian | 391,349 (55.62%) | 312,217 (44.38%) |
|  | White British vs. African | 276,134 (55.79%) | 218,850 (44.21%) |
| **Total-cholesterol** | White British vs. Other European | 462,719 (51.99%) | 427,313 (48.01%) |
|  | White British vs. South Asian | 366,856 (52.14%) | 336,710 (47.86%) |
|  | White British vs. African | 259,545 (52.44%) | 235,439 (47.66%) |
| **HDL-cholesterol** | White British vs. Other European | 462,579 (51.97%) | 427,453 (48.03%) |
|  | White British vs. South Asian | 366,202 (52.05%) | 337,364 (47.95%) |
|  | White British vs. African | 258,200 (52.16%) | 236,784 47.84%) |
| **LDL-cholesterol** | White British vs. Other European | 456,663 (51.31%) | 433,369 (48.69%) |
|  | White British vs. South Asian | 361,860 (51.43%) | 341,706 (48.57%) |
|  | White British vs. African | 255,268 (51.57%) | 239,716 (48.43%) |

**Table S3: P-value for the significant difference between each pair of the methods across ancestries for BMI while using 50,000 white British as discovery.**

| **Methods** | **GBLUP** | **PLINK** | **PolyPred** | **PRS-CSx** | **PRSice** | **XPASS** |
| --- | --- | --- | --- | --- | --- | --- |
|  | **Other European** | | | | | |
| **Bolt-LMM** | 2.49e-11 | 4.62e-01 | 7.01e-01 | 9.89e-01 | 7.33e-06 | 9.68e-06 |
| **GBLUP** |  | 3.73e-11 | 5.70e-05 | 3.51e-07 | 9.85e-16 | 2.22e-04 |
| **PLINK** |  |  | 8.80e-01 | 8.47e-01 | 7.37e-06 | 3.58e-09 |
| **PolyPred** |  |  |  | 7.99e-01 | 2.02e-04 | 7.5e-03 |
| **PRS-CSx** |  |  |  |  | 7.20e-10 | 1.75e-04 |
| **PRSice** |  |  |  |  |  | 1.57e-13 |
| **XPASS** |  |  |  |  |  |  |
|  | **South Asian** | | | | | |
| **Bolt-LMM** | 1.20e-02 | 3.50e-01 | 3.00e-01 | 7.87e-01 | 1.5e-03 | 1.71e-01 |
| **GBLUP** |  | 4.81e-02 | 2.11e-02 | 3.71e-02 | 1.34e-05 | 1.32e-01 |
| **PLINK** |  |  | 2.02e-01 | 5.82e-01 | 7.02e-04 | 2.69e-01 |
| **PolyPred** |  |  |  | 6.21e-01 | 1.89e-02 | 1.01e-01 |
| **PRS-CSx** |  |  |  |  | 1.11e-04 | 1.09e-01 |
| **PRSice** |  |  |  |  |  | 3.47e-05 |
|  | **African** | | | | | |
| **Bolt-LMM** | 4.31e-01 | 7.04e-01 | 1.53e-01 | 8.16e-01 | 5.45e-01 | 6.77e-01 |
| **GBLUP** |  | 5.41e-01 | 4.31e-01 | 3.99e-01 | 2.88e-01 | 6.17e-01 |
| **PLINK** |  |  | 7.04e-01 | 7.23e-01 | 4.89e-01 | 7.92e-01 |
| **PolyPred** |  |  |  | 8.16e-01 | 5.45e-01 | 6.76e-01 |
| **PRS-CSx** |  |  |  |  | 5.90e-01 | 4.69e-01 |
| **PRSice** |  |  |  |  |  | 3.82e-01 |

P-values were estimated using an R-package (r2redux)^1^.

**Table S4: P-value for the significant difference between each pair of the methods across ancestries for standing height while using 50,000 white British as discovery.**

| **Methods** | **GBLUP** | **PLINK** | **PolyPred** | **PRS-CSx** | **PRSice** | **XPASS** |
| --- | --- | --- | --- | --- | --- | --- |
|  | **Other European** | | | | | |
| **Bolt-LMM** | 9.13e-51 | 2.04e-05 | 2.16e-22 | 1.08e-18 | 3.16e-03 | 8.79e-11 |
| **GBLUP** |  | 2.76e-53 | 8.37e-01 | 6.09e-02 | 6.69e-07 | 1.69e-16 |
| **PLINK** |  |  | 1.01e-27 | 1.85e-31 | 1.04e-05 | 3.31e-23 |
| **PolyPred** |  |  |  | 4.23e-01 | 2.04e-06 | 3.87-04 |
| **PRS-CSx** |  |  |  |  | 1.97e-05 | 2.04e-25 |
| **PRSice** |  |  |  |  |  | 1.48e-01 |
|  | **South Asian** | | | | | |
| **Bolt-LMM** | 7.33e-05 | 1.34e-01 | 2.01e-05 | 3.49e-02 | 6.20e-01 | 1.97e-01 |
| **GBLUP** |  | 1.36e-05 | 9.03e-01 | 2.33e-01 | 1.46e-02 | 1.27e-03 |
| **PLINK** |  |  | 5.06e-06 | 4.13e-03 | 9.05e-01 | 3.14e-02 |
| **PolyPred** |  |  |  | 5.16e-01 | 4.18e-03 | 9.63e-02 |
| **PRS-CSx** |  |  |  |  | 5.46e-02 | 2.62e-03 |
| **PRSice** |  |  |  |  |  | 2.22e-01 |
|  | **African** | | | | | |
| **Bolt-LMM** | 1.58e-05 | 5.08e-01 | 2.40e-03 | 1.34e-02 | 1.80e-02 | 1.06e-01 |
| **GBLUP** |  | 1.93e-06 | 5.44e-01 | 8.21e-01 | 7.28e-01 | 1.75e-04 |
| **PLINK** |  |  | 2.49e-04 | 5.02e-03 | 4.85e-03 | 9.10e-03 |
| **PolyPred** |  |  |  | 9.21e-01 | 8.71e-01 | 1.32e-01 |
| **PRS-CSx** |  |  |  |  | 8.72e-01 | 1.92e-01 |
| **PRSice** |  |  |  |  |  | 1.74e-01 |

P-values were estimated using an R-package (r2redux)^1^.

**Table S5: P-value for the significant difference between each pair of the methods across ancestries for total cholesterol while using 50,000 white British as discovery.**

| **Methods** | **GBLUP** | **PLINK** | **PolyPred** | **PRS-CSx** | **PRSice** | **XPASS** |
| --- | --- | --- | --- | --- | --- | --- |
|  | **Other European** | | | | | |
| **Bolt-LMM** | 2.06e-03 | 5.33e-02 | 4.51e-17 | 3.97e-10 | 1.95e-23 | 1.34e-04 |
| **GBLUP** |  | 6.66e-02 | 9.14e-15 | 7.36e-11 | 6.30e-22 | 1.04e-01 |
| **PLINK** |  |  | 3.27e-16 | 1.47e-09 | 8.09e-23 | 8.34e-04 |
| **PolyPred** |  |  |  | 1.90e-04 | 3.12e-08 | 5.24e-14 |
| **PRS-CSx** |  |  |  |  | 7.10e-14 | 2.18e-10 |
| **PRSice** |  |  |  |  |  | 2.68e-21 |
|  | **South Asian** | | | | | |
| **Bolt-LMM** | 7.97e-02 | 8.5e-01 | 8.09e-06 | 4.03e-02 | 1.61e-04 | 2.29e-01 |
| **GBLUP** |  | 1.09e-01 | 3.30e-05 | 1.11e-01 | 4.87e-04 | 3.29e-01 |
| **PLINK** |  |  | 8.79e-06 | 4.07e-02 | 1.68e-04 | 2.16e-01 |
| **PolyPred** |  |  |  | 6.36e-04 | 9.83e-01 | 2.06e-05 |
| **PRS-CSx** |  |  |  |  | 2.89e-03 | 3.45e-02 |
| **PRSice** |  |  |  |  |  | 3.11e-04 |
|  | **African** | | | | | |
| **Bolt-LMM** | 2.28e-01 | 8.79e-01 | 3.15e-04 | 2.13e-02 | 2.96e-03 | 6.24e-01 |
| **GBLUP** |  | 2.53e-01 | 1.58e-02 | 7.61e-02 | 6.74e-02 | 3.28e-01 |
| **PLINK** |  |  | 3.77e-04 | 2.13e-02 | 3.03e-02 | 6.44e-01 |
| **PolyPred** |  |  |  | 7.95e-01 | 4.10e-01 | 1.08e-03 |
| **PRS-CSx** |  |  |  |  | 3.39e-01 | 7.22e-03 |
| **PRSice** |  |  |  |  |  | 03.72e-02 |

P-values were estimated using an R-package (r2redux)^1^.

**Table S6:** **P values to test the significance of difference between methods across ancestries for HDL-cholesterol while using 50,000 white British as discovery.**

| **Methods** | **GBLUP** | **PLINK** | **PolyPred** | **PRS-CSx** | **PRSice** | **XPASS** |
| --- | --- | --- | --- | --- | --- | --- |
|  | **Other European** | | | | | |
| **Bolt-LMM** | 9.23e-09 | 1.11e-02 | 9.12e-51 | 8.68e-11 | 1.05e-32 | 1.88e-03 |
| **GBLUP** |  | 7.63e-11 | 4.43e-40 | 6.34e-12 | 2.99e-26 | 3.81e-04 |
| **PLINK** |  |  | 1.28e-52 | 1.26e-08 | 2.44e-34 | 1.60e-08 |
| **PolyPred** |  |  |  | 2.48e-05 | 1.72e-01 | 7.34e-44 |
| **PRS-CSx** |  |  |  |  | 7.23e-25 | 9.89e-20 |
| **PRSice** |  |  |  |  |  | 6.56e-29 |
|  | **South Asian** | | | | | |
| **Bolt-LMM** | 7.79e-03 | 7.59e-01 | 1.10e-17 | 1.21e-01 | 7.02e-16 | 1.09e-02 |
| **GBLUP** |  | 1.91e-02 | 1.35e-12 | 1.11e-02 | 2.19e-13 | 7.15e-01 |
| **PLINK** |  |  | 8.47e-17 | 1.07e-01 | 9.04e-16 | 5.46e-03 |
| **PolyPred** |  |  |  | 6.51e-03 | 1.14e-05 | 3.77e-13 |
| **PRS-CSx** |  |  |  |  | 3.35e-05 | 1.02e-02 |
| **PRSice** |  |  |  |  |  | 9.17e-14 |
|  | **African** | | | | | |
| **Bolt-LMM** | 5.12e-02 | 5.70e-01 | 1.56e-04 | 7.84e-02 | 9.97e-07 | 9.78e-02 |
| **GBLUP** |  | 4.09e-02 | 1.82e-03 | 8.94e-03 | 1.97e-05 | 2.22e-01 |
| **PLINK** |  |  | 1.23e-04 | 9.20e-02 | 7.59e-07 | 3.68e-02 |
| **PolyPred** |  |  |  | 2.76e-02 | 6.59e-02 | 8.58e-04 |
| **PRS-CSx** |  |  |  |  | 3.50e-02 | 1.24e-02 |
| **PRSice** |  |  |  |  |  | 9.54e-06 |

P-values were estimated using an R-package (r2redux)^1^.

**Table S7:** **P values to test the significance of difference between methods across ancestries for LDL-cholesterol while using 50,000 white British as discovery**

| **Methods** | **GBLUP** | **PLINK** | **PolyPred** | **PRS-CSx** | **PRSice** | **XPASS** |
| --- | --- | --- | --- | --- | --- | --- |
|  | **Other European** | | | | | |
| **Bolt-LMM** | 7.37e-03 | 3.37e-01 | 8.67e-11 | 2.54e-09 | 5.02e-32 | 6.10e-04 |
| **GBLUP** |  | 5.39e-01 | 9.05e-10 | 5.32e-10 | 6.43e-31 | 1.58e-01 |
| **PLINK** |  |  | 1.49e-10 | 3.84e-09 | 9.19e-32 | 5.48e-04 |
| **PolyPred** |  |  |  | 4.34e-03 | 1.53e-18 | 2.15e-09 |
| **PRS-CSx** |  |  |  |  | 1.19e-22 | 1.41e-09 |
| **PRSice** |  |  |  |  |  | 2.05e-30 |
|  | **South Asian** | | | | | |
| **Bolt-LMM** | 4.59e-01 | 9.61e-01 | 8.54e-06 | 1.67e-02 | 3.04e-04 | 5.99e-01 |
| **GBLUP** |  | 4.70e-01 | 1.45e-05 | 1.73e-02 | 4.34e-04 | 7.23e-01 |
| **PLINK** |  |  | 8.86e-06 | 1.59e-02 | 3.03e-04 | 5.33e-01 |
| **PolyPred** |  |  |  | 2.64e-03 | 5.84e-01 | 1.26e-05 |
| **PRS-CSx** |  |  |  |  | 7.21e-03 | 8.07e-03 |
| **PRSice** |  |  |  |  |  | 3.80e-04 |
|  | **African** | | | | | |
| **Bolt-LMM** | 1.50e-01 | 8.94e-01 | 2.52e-04 | 6.23e-03 | 9.71e-03 | 8.77e-01 |
| **GBLUP** |  | 1.49e-01 | 7.44e-04 | 1.88e-02 | 2.57e-02 | 1.30e-01 |
| **PLINK** |  |  | 2.55e-04 | 6.02e-03 | 9.59e-03 | 9.12e-01 |
| **PolyPred** |  |  |  | 9.82e-02 | 8.78e-01 | 2.98e-04 |
| **PRS-CSx** |  |  |  |  | 3.12e-01 | 2.92e-03 |
| **PRSice** |  |  |  |  |  | 9.06e-03 |

P-values were estimated using an R-package (r2redux)^1^.

**Supplementary Reference**

1. Momin, M.M., et al., *Significance tests for R2 of out-of-sample prediction using polygenic scores.* The American Journal of Human Genetics, 2023. **110**: p. 349-358.
